# GPU-Accelerated Finite Element Method for Modelling Light Transport in Diffuse Optical Tomography

**DOI:** 10.1155/2011/403892

**Published:** 2011-10-16

**Authors:** Martin Schweiger

**Affiliations:** Department of Computer Science, University College London, Gower Street, London WC1E 6BT, UK

## Abstract

We introduce a GPU-accelerated finite element forward solver for the computation of light transport in scattering media. The forward model is the computationally most expensive component of iterative methods for image reconstruction in diffuse optical tomography, and performance optimisation of the forward solver is therefore crucial for improving the efficiency of the solution of the inverse problem. The GPU forward solver uses a CUDA implementation that evaluates on the graphics hardware the sparse linear system arising in the finite element formulation of the diffusion equation. We present solutions for both time-domain and frequency-domain problems. A comparison with a CPU-based implementation shows significant performance gains of the graphics accelerated solution, with improvements of approximately a factor of 10 for double-precision computations, and factors beyond 20 for single-precision computations. The gains are also shown to be dependent on the mesh complexity, where the largest gains are achieved for high mesh resolutions.

## 1. Introduction

Diffuse optical tomography (DOT) is a functional imaging modality for medical applications that has the potential to provide three-dimensional images of the scattering and absorption parameter distributions *in vivo*, from which clinically relevant physiological parameters such as tissue and blood oxygenation states and state changes can be derived. Applications include brain activation visualisation [1, 2], brain oxygenation monitoring in infants [3], and breast tumour detection [4].

Data acquisition systems consist of an infrared light delivery system that illuminates the tissue surface at different locations, and detectors that measure the transmitted light at a set of surface positions. Measurements can be performed in continuous wave (CW) mode, in time-resolved mode using ultra-short input pulses and time-resolved detectors, or in frequency-domain mode, using modulated light sources and measuring the phase shift and modulation amplitude at the detector locations.

Due to the high level of scattering in most biological tissues, image reconstruction in DOT is an ill-posed nonlinear problem whose solution generally requires the formulation of a forward model of light propagation in inhomogeneous scattering tissue. Frequently utilised light transport models include stochastic models such as Monte-Carlo simulation [5], or deterministic models such as the radiative transfer equation (RTE) [6] or the diffusion equation (DE) [7]. Numerical solution approaches include finite difference, finite element, finite volume, or boundary element methods. The light transport model considered in this paper the finite element method (FEM) for the solution of the diffusion equation. The reconstruction problem can be stated as a nonlinear optimisation problem, where an objective function, defined as a norm of the difference between measurement data and model data for a given set of optical parameters, is minimised, subject to a regularisation functional. Reconstruction approaches include methods that require the availability of the forward model only, such as Markov-Chain Monte-Carlo methods, its first derivative, such as nonlinear conjugate gradient methods, and its second derivative, such as Newton-type methods.

Iterative solvers require multiple evaluations of the forward model for calculating the objective function and its gradient. The forward model itself involves the solution of a large linear system with multiple right-hand sides. Problems involving high-dimensional parameter spaces result in time-consuming evaluations of the forward model, which limits the applicability of the reconstruction methods in clinical practice. Significant performance improvements are required to make DOT a viable tool in medical imaging. Recent developments in computing hardware have offered the possibility to make use of parallel computation. Traditionally, solutions have included central processing unit (CPU) based moderately parallel systems with shared memory access (multiprocessor and multicore implementation) and large-scale distributed parallel systems limited by data transfer between nodes (cluster CPU implementation). More recently, the parallel architecture of graphics processing units (GPU) has been utilised for the acceleration of general purpose computations, including GPU methods for the solution of dense [8, 9] or sparse [10–14] linear systems. The latter are encountered in the implementation of the FEM.

In the context of diffuse optical tomography and related fields of optical imaging, GPU-accelerated computations have been successfully employed for implementing Monte-Carlo light transport models [15–17], which compute independent photon trajectories and are well-suited for parallelisation due to the lack of interprocess communication. Acceleration rates of more than 300 are possible. Zhang et al. [18] have applied GPU acceleration to finite element computations in bioluminescence tomography and compared to single and multithreaded CPU performance. They reported significant performance advantages of the GPU version but were limited to low mesh complexity due to memory limits. In optical projection tomography, GPU-based reconstruction methods have been employed by Vinegoni et al. [19]. Watanabe and Itagaki [20] have used a GPU implementation for real-time visualisation in Fourier-domain optical coherence tomography.

In this paper, we are investigating the potential of a GPU implementation for the forward model in DOT. We present a Compute Unified Device Architecture (CUDA) version of the finite element forward solver presented previously [21, 22], using the CUSP library [23] for sparse linear system computation on the graphics processor. CUDA is the computing architecture for NVidia graphics processors and can be addressed via an application-programming interface (API). Current GPU hardware is performance optimised for single-precision arithmetic. We investigate the effect of single-precision computation on the accuracy of the forward model for different combinations of optical parameters. We compare the performance of the GPU forward solver with an equivalent CPU implementation. We show that significant performance improvements can be achieved. The evaluation of the forward model is the most time-consuming element of iterative inverse solvers, and any efficiency gains in the forward solver therefore directly translate into reduced overall runtimes for image reconstruction applications and are an important step towards making DOT a viable imaging application in clinical practice.

## 2. Methodology

### 2.1. Finite Element Solver

We consider the diffusion approximation to the radiative transfer equation [24, 25] in either steady-state, time, or frequency domain as the forward model for light transport in tissue. For steady-state problems, the stationary real-valued photon density inside the medium arising from a continuous-wave source is computed while for frequency-domain problems, the source is amplitude modulated, giving rise to a complex-valued solution of a photon density wave distribution. In time-domain problems, the source is considered a delta-pulse in time, and the measurement consists of the temporal dispersion of the transmitted signal. Given a compact domain *Ω* bounded by ∂*Ω*, the diffusion equation [26] in time and frequency domain is given by

(1) $$\begin{array}{c} \begin{array}{c} \left[-\nabla \cdot \kappa \left(r\right)\nabla +\mu_{a}\left(r\right)+\frac{1}{c}\frac{\partial }{\partial t}\right]\phi \left(r,t\right)=0\\ \left[-\nabla \cdot \kappa \left(r\right)\nabla +\mu_{a}\left(r\right)+\frac{i\omega }{c}\right]\hat{\phi }\left(r,\omega \right)=0 \end{array}\}r\in \Omega , \end{array}$$

respectively, where *ω* is the angular source modulation frequency, *κ*(**r**)  and  *μ*_*a*_(**r**) are the spatially varying diffusion and absorption coefficients, respectively, where *κ* = [3(*μ*_*a*_ + *μ*_*s*_)]^−1^ with scattering coefficient *μ*_*s*_. *c* is the speed of light in the medium, and $\phi ,\;\text{and}\;\hat{\phi }$ are the real and complex-valued photon density fields. For simplicity in the following, we use *ϕ* to denote either the real or complex-valued properties as appropriate.

A Robin-type boundary condition [27] applies at ∂*Ω*,

(2) $$\phi \left(\xi \right)+2\zeta \left(n\right)\kappa \left(\xi \right)\frac{\partial \phi }{\partial \nu }=q\left(\xi \right),\;\xi \in \partial \Omega ,$$

where *q* is a real or complex-valued source distribution as appropriate, *ζ*(*n*) is a boundary reflectance term incorporating the refractive index *n* at the tissue-air interface, and *ν* is the surface normal at surface point *ξ*. The boundary operator defining the exitance Γ through ∂*Ω* is given by the Dirichlet-to-Neumann map

(3) $$\Gamma \left(\xi \right)=-c\kappa \left(\xi \right)\frac{\partial \phi }{\partial \nu }=\frac{c}{2\zeta }\phi \left(\xi \right).$$

The set of measurements *y*_*ij*_ from a source distribution *q*_*i*_ is obtained by integrating Γ over the measurement profiles *m*_*j*_(*ξ*) on the surface

(4) $$y_{ij}=\int_{\partial \Omega }\Gamma_{i}\left(\xi \right)m_{j}\left(\xi \right)d\xi .$$

For the time-domain problem, *y*_*ij*_ are the temporal dispersion profiles of the received signal intensities while, for the frequency-domain problem, *y*_*ij*_ are given by the complex exitance values, usually expressed by logarithmic amplitude ln⁡*A* and phase shift *φ* [28],

(5) ln⁡Aij=Re(ln⁡yij),  φij=Im⁡(ln⁡yij).

Given the set of forward data **y** = {*y*_*ij*_} of all measurements from all source distributions, (1) to (4) define the forward model *f*[*κ*, *μ*_*a*_] = **y** which maps a parameter distribution *κ*, *μ*_*a*_ to measurements for a given domain geometry, modulation frequency, source distributions, and measurement profiles.

The forward model is solved numerically by using a finite element approach. A division of domain *Ω* into tetrahedral elements defined by *N* vertex nodes provides a piecewise polynomial basis for the parameters *κ*, *μ*_*a*_, and photon density *ϕ*. The approximate field *ϕ*^*h*^(**r**) at any point **r** ∈ *Ω* is given by interpolation of the nodal coefficients *ϕ*_*i*_ using piecewise polynomial shape functions *u*_*i*_(**r**)

(6) $$\phi^{h}\left(r\right)=\overset{N}{\underset{i=1}{\sum }}u_{i}\left(r\right)\phi_{i}.$$

Piecewise polynomial approximations *κ*^*h*^, *μ*_*a*_^*h*^ to the continuous parameters, defined by the nodal coefficients *κ*_*i*_, *μ*_*a*,*i*_ are constructed in the same way. Applying a Galerkin approach transforms the continuous problem of (1) into an *N*-dimensional discrete problem of finding the nodal field values Φ = {*ϕ*_*i*_} at all nodes *i*, given the set of nodal parameters **x** = {*κ*_*i*_, *μ*_*a*,*i*_}. For the frequency-domain problem, the resulting linear system is given by

(7) S(x,ω)Φ(ω)=Q(ω),

where

(8) $$S\left(x,\omega \right)=K\left(\left\{\kappa_{i}\right\}\right)+C\left(\left\{\mu_{a,i}\right\}\right)+\gamma A+i\omega B,$$

*γ* = *c*/2*ζ*, *K*, *C*, *A*, *B* ∈ ℝ^*N*×*N*^ are symmetric sparse matrices given by [7]

(9) $$\begin{array}{cc} K_{ij} & =\overset{N}{\underset{k=1}{\sum }}\kappa_{k}\int_{\Omega }u_{k}\left(r\right)\nabla u_{i}\left(r\right)\cdot \nabla u_{j}\left(r\right)dr,\\ C_{ij} & =\overset{N}{\underset{k=1}{\sum }}\mu_{a,k}\int_{\Omega }u_{k}\left(r\right)u_{i}\left(r\right)u_{j}\left(r\right)dr,\\ A_{ij} & =\int_{\partial \Omega }u_{i}\left(\xi \right)u_{j}\left(\xi \right)d\xi ,\\ B_{ij} & =\frac{1}{c}\int_{\Omega }u_{i}\left(r\right)u_{j}\left(r\right)dr. \end{array}$$

And right-hand side *Q* is given by

(10) $$Q_{i}=\overset{N}{\underset{k=1}{\sum }}q_{i}\int_{\partial \Omega }u_{i}\left(\xi \right)d\xi$$

with *q*_*i*_ the nodal coefficients of the basis expansion of *q*(**ξ**). For the solution of the time-domain problem, the time derivative in (1) at time *t* is approximated by a finite difference

(11) $$\begin{array}{c} \frac{\partial \phi \left(\vec{r},t\right)}{\partial t}\approx \frac{1}{\Delta t}\left[\phi \left(\vec{r},t+\Delta t\right)-\phi \left(\vec{r},t\right)\right]. \end{array}$$

The temporal profile of *ϕ* is approximated at a set of discrete steps {*t*_*n*_} and evaluated by a finite difference approach, given by the iteration

(12) $$\begin{array}{cc} \left[\theta \tilde{S}+\frac{1}{\Delta t_{0}}B\right]\Phi \left(t_{0}\right) & =\frac{1}{\Delta t_{0}}Q_{0},\\ \left[\theta \tilde{S}+\frac{1}{\Delta t_{n}}B\right]\Phi \left(t_{n}\right) & =-\left[\left(1-\theta \right)\tilde{S}-\frac{1}{\Delta t_{n}}B\right]\Phi \left(t_{n-1}\right),\;n\geq 1, \end{array}$$

where $\tilde{S}=K+C+\gamma A$, time steps *t*_*n*_ = *t*_*n*−1_ + Δ*t*_*n*−1_, *n* ≥ 1, and 0 ≤ *θ* ≤ 1 is a control parameter that can be used to select implicit (*θ* = 1), explicit (*θ* = 0), or intermediate schemes. The step lengths Δ*t*_*n*_ are governed by stability considerations of the finite difference scheme. For the unconditionally stable implicit scheme, the step length can be adjusted to the curvature of the temporal profile, allowing increased step length at the exponentially decaying tail of *ϕ*(*t*).

The solution of the FEM problem thus consists of (i) construction of the system matrices (9), (ii) solution of the complex-valued linear problem (7) or real-valued sequence of linear problems (12), and (iii) mapping to measurements (3) and (4). The main computational cost is the solution of the linear system, in particular in the time-domain problem, while the cost of matrix assembly time is typically only 1–10% of the time of a single linear solution. The linear system can be solved either with a direct method, such as Cholesky decomposition for the real-valued time-domain problem or LU decomposition for the complex-valued frequency domain problem, or with iterative methods, such as conjugate gradients for the real-valued problem and biconjugate gradients for the complex-valued problem. Direct methods become impractical for large-scale problems, due to memory storage requirements for the decomposition and increased computation time. For 3-D problems with high node density, iterative solvers are generally employed.

### 2.2. GPU Implementation

The bottleneck of the reconstruction problem is the solution of the linear systems in (7) or (12). Accelerating the linear solver is therefore an effective method for improving the inverse solver performance. We have embedded a graphics processor-accelerated version of the FEM forward solver into the existing TOAST software package [29] for light transport and reconstruction presented previously [7]. The GPU-accelerated code uses the CUDA programming interface for NVidia graphics processor hardware. The implementation utilises the CUSP library which offers a templated framework for sparse linear algebra and provides conjugate gradient (CG) and biconjugate gradient-stabilised (BiCGSTAB) iterative solvers for sparse linear systems. The library supports both single and double precision computation if supported by hardware.

We use the compressed sparse row (CSR) format for matrix storage. There are alternative storage formats such as the coordinate, ELLPACK, or hybrid formats [11] which can provide better parallel performance depending on the matrix fill structure, usually at the cost of less compact storage. However, the CSR format constitutes a good compromise between performance and versatility and is well suited for the matrix fill distribution arising from unstructured FEM meshes.

For the solution of the complex-valued linear problem (7), we expand the complex *N* × *N* system into a 2*N* × 2*N* real system of the form

(13) $$\left[\begin{array}{cc} S_{\text{re}} & -S_{\text{im}}\\ S_{\text{im}} & S_{\text{re}} \end{array}\right]\left[\begin{array}{c} \Phi_{\text{re}}\\ \Phi_{\text{im}} \end{array}\right]=\left[\begin{array}{c} Q_{\text{re}}\\ Q_{\text{im}} \end{array}\right].$$

The CUSP CG and BiCGSTAB solvers had to be modified to account for early termination of the iteration loop due to singularities in the intermediate results. Early termination conditions occasionally do occur in practice in the problems considered in this paper, in particular due to single-precision round-off errors.

**Figure 1:**
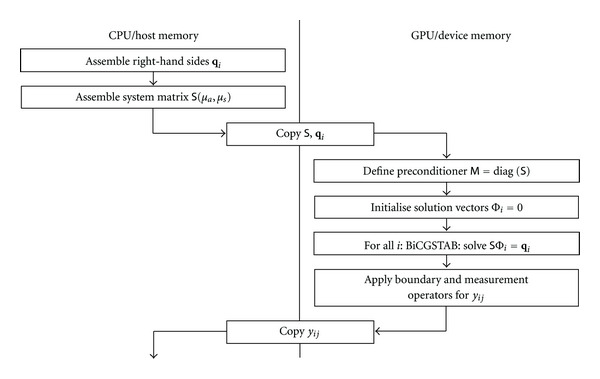
Data flow between host and graphics device for solution of linear problem (7).

The data flow between host and graphics device memory for a single solver step is shown in Figure 1. The system matrix *S* is assembled in host memory for a given set of parameters, together with the source vectors **q**_*i*_, and copied to GPU device memory. The GPU solver is then invoked for all right-hand-sides, after which the projected solutions *y*_*ij*_ are copied back to host memory.

**Figure 2:**
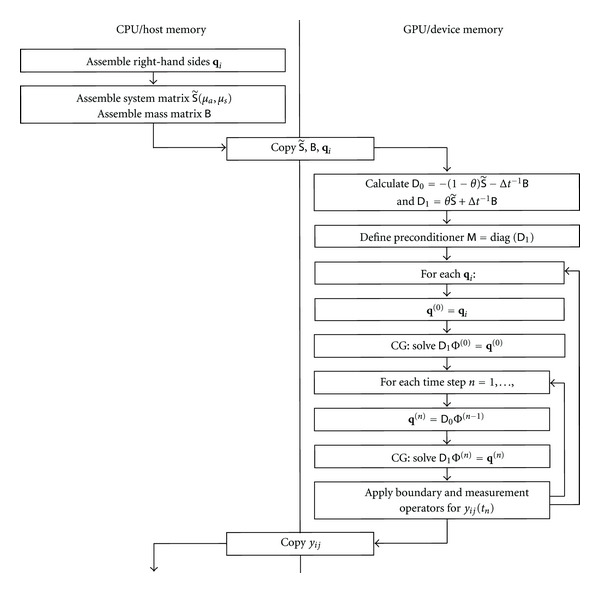
Data flow between host and graphics device for solution of linear problem (12).

For the finite-difference solution of the time-domain problem, the entire iteration (12) can be evaluated on the GPU with minimal communication between host and graphics system, consisting of initial copying the system matrices $\tilde{S}$ and *B* to the GPU, and returning the computed temporal profiles *y*_*ij*_(*t*) back to the host. The data flow diagram for the time-domain problem is shown in Figure 2.

### 2.3. Single-Precision Arithmetic

GPU hardware is traditionally optimised for single-precision floating point operations. Although GPU hardware with double-precision capability is emerging, typically only a fraction of the chip infrastructure is dedicated to double-precision operations, thus incurring a significant performance penalty. For optimising, it is therefore advantageous to use single-precision arithmetic where adequate. We have implemented the FEM solver in both single and double precision for GPU as well as CPU platforms.

When the system matrix is represented in single precision, care has to be taken during assembly. The system matrix is assembled from individual element contributions (9). The global vertex contributions in the system matrix are the sum of the local element vertex values for all elements adjacent to the vertex. During the summation, loss of precision can occur if the magnitude difference between the summands is large compared to the mantissa precision of the floating point representation. For single precision arithmetic, this can be a problem in particular where vertices have a large number of adjacent elements, notably in 3-D meshes with tetrahedral elements. Loss of precision during matrix assembly can be reduced if the contributions are sorted from smallest to highest magnitude. However, this incurs a book-keeping overhead that can impact on performance. Instead we have opted to assemble the system matrix in double precision and map the values to single precision after assembly. The assembly step is performed on the host side, with negligible performance impact because assembly time is generally small compared to solve time.

To compare the results of matrix assembly in single and double precision, we have performed an FEM forward solution from single-precision system matrices that were assembled in both single and double precision. The domain was a homogeneous cylinder of radius 25 mm and height 50 mm, with optical parameters *μ*_*a*_ = 0.01 mm^−1^ and *κ* = 0.3 mm. A point source modulated at frequency *ω* = 2*π* · 100 MHz was placed on the cylinder mantle. The mesh consisted of 83142 and 444278 tetrahedral elements.

**Figure 3:**
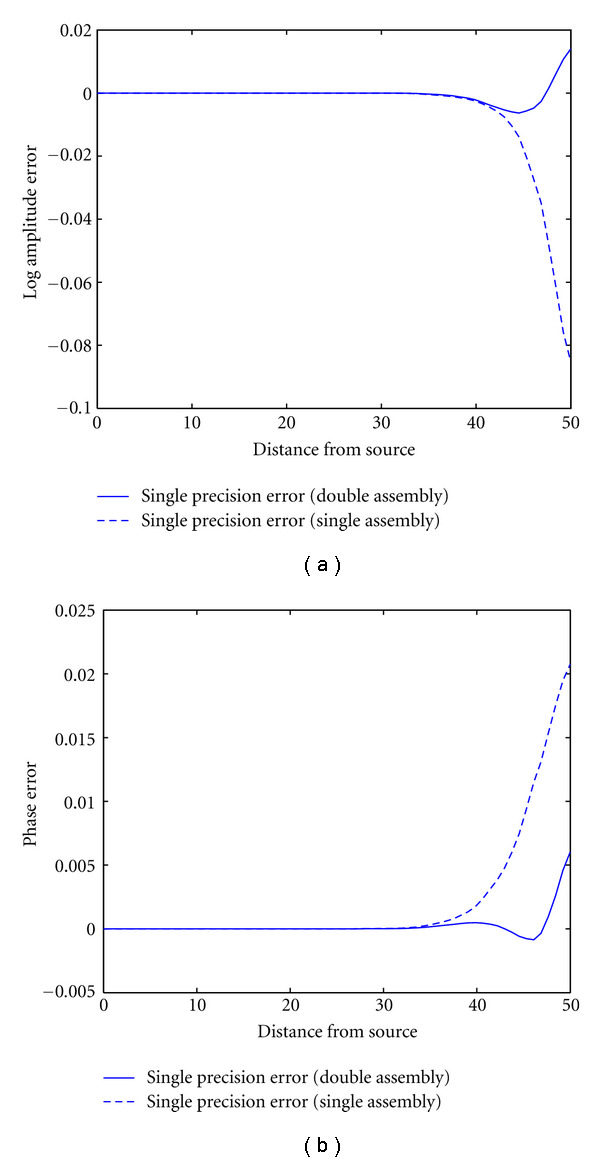
Effect of single-precision arithmetic on forward solutions. Shown are the differences between single and double precision solutions for logarithmic amplitude (a) and phase (b) of the complex field computed in a cylindrical domain along a line from the source across the cylinder. The solid line shows the solution error for a system matrix assembled in double precision and solved in single precision while the dashed line represents the solution for a system matrix assembled and solved in single precision.

Figure 3 shows the differences between single and double precision forward solution in log amplitude (Figure 3(a)) and phase (Figure 3(b)) of the complex photon density field along a line from the source position across the volume of the cylinder. The solid lines represent the single-precision error where the system matrix has been assembled in double precision before being mapped to single precision, while the dashed line is the error arising from a system matrix assembled in single precision. It can be seen that system matrix assembly in double precision can significantly reduce the solution errors, in particular at large distances from the source.

**Figure 4:**
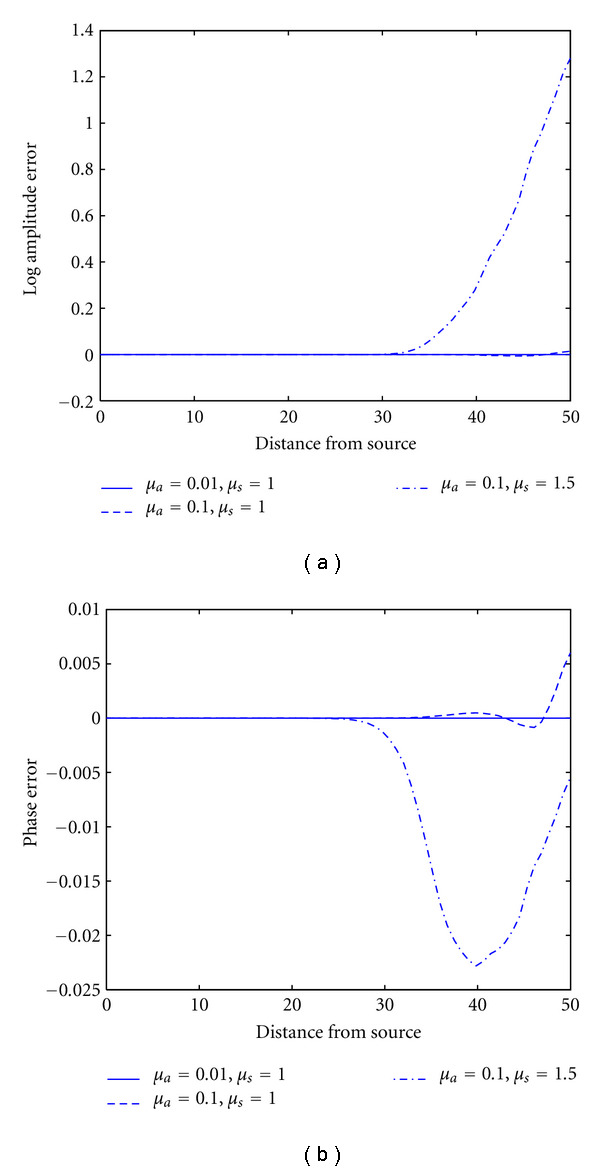
Single-precision arithmetic error as a function of optical coefficients. Shown are the differences between single and double precision results in log amplitude (a) and phase (b) along a line from the source across the cylinder, for three different combinations of absorption and scattering parameters.

The influence of optical parameters on the single-precision error of the forward data is shown in Figure 4. The forward solutions were calculated for three different combinations of absorption and scattering coefficient (i) *μ*_*a*_ = 0.01 mm^−1^, *μ*_*s*_ = 1 mm^−1^, (ii) *μ*_*a*_ = 0.1 mm^−1^, *μ*_*s*_ = 1 mm^−1^, and (iii) *μ*_*a*_ = 0.1 mm^−1^, *μ*_*s*_ = 1.5 mm^−1^. It can be seen that the discrepancies become more severe at higher values of the optical parameters. The results are particularly sensitive to an increase of the scattering parameter. Due to attenuation, the photon density fields inside the object decay rapidly, leading to large dynamic range in the data. Increased absorption and scattering parameters aggravate this effect, which impairs the accuracy of the single-precision solution, in particular in regions far away from the source. It should be noted, however, that, for moderate optical parameters in a typical range for optical tomography, the single precision solution is accurate, with maximum relative errors of 10^−6^ to 10^−4^ in log amplitude and phase, respectively.

## 3. Results

**Figure 5:**
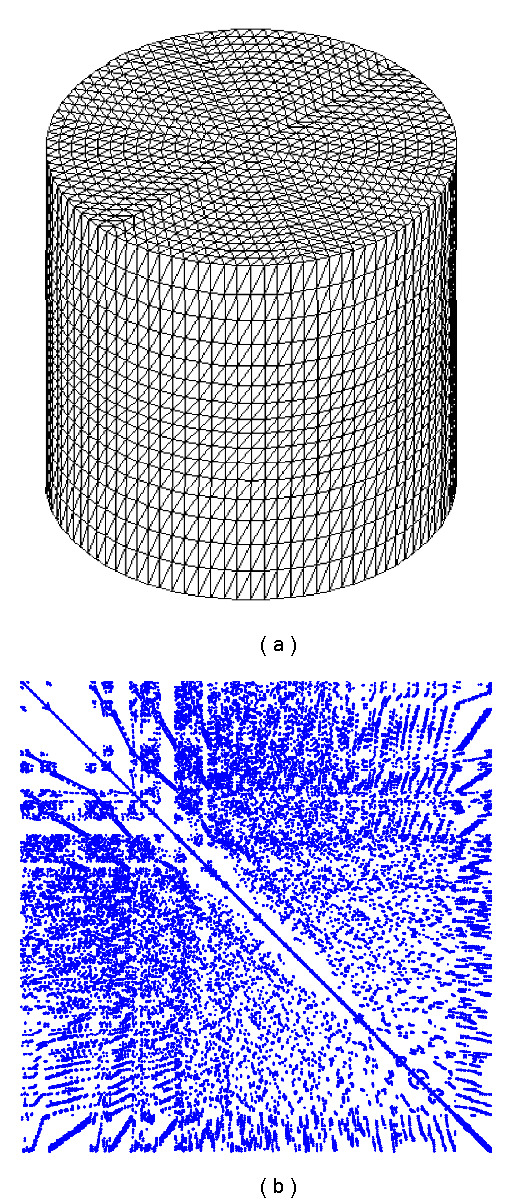
Cylinder geometry for the forward and inverse solver problems, showing a mesh with 83142 nodes and 444278 tetrahedral elements (b). The fill structure of the resulting FEM system matrix is shown on the right. The number of nonzeros is 1150264, resulting in a fill fraction of 1.664 · 10^−4^.

The graphics accelerated forward solver problems were executed on an NVidia GTX 285 GPU. The technical specifications of the device are listed in Table 1. The device supports double as well as single precision arithmetic, so results for both were collected. For performance comparison, the same model calculations were also performed with a CPU-based serial implementation on an Intel Xeon processor clocked at 2.0 GHz with 4 MB cache and 12 GB main memory. The FEM model consisted of a homogeneous cylindrical mesh with radius 25 mm and height 50 mm at various element resolutions. 80 sources and 80 detectors were arranged on the surface. One of the meshes, together with the resulting system matrix sparsity structure, is shown in Figure 5. 

### 3.1. Frequency Domain Solver

**Figure 6:**
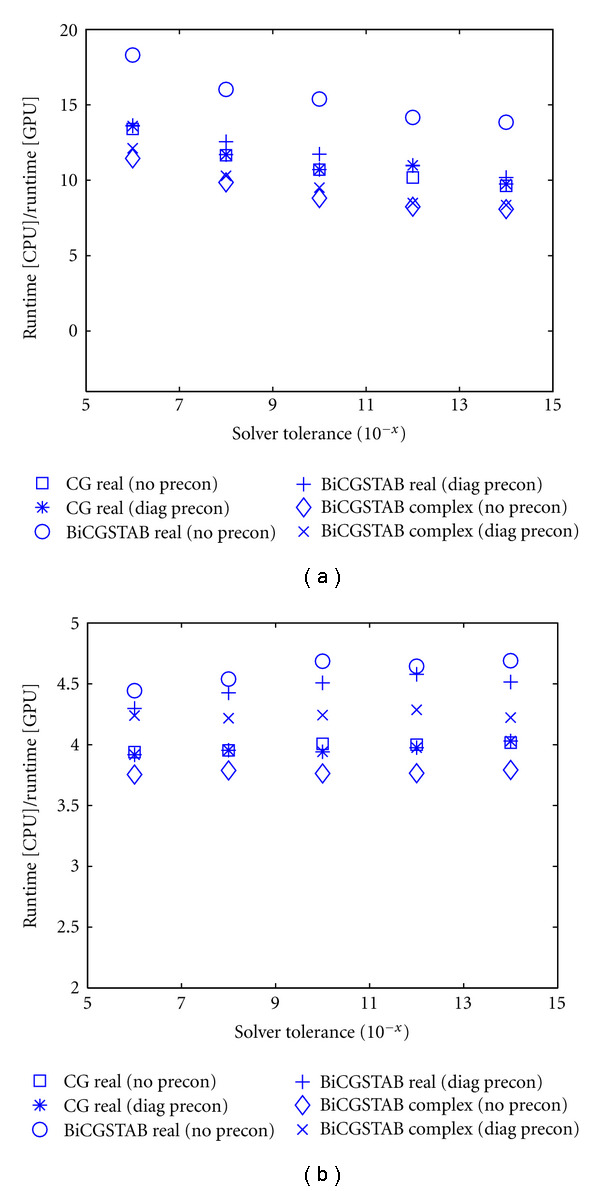
GPU performance factor as a function of linear solver tolerance for real and complex problems, using CG and BiCGSTAB solvers, without preconditioner and with diagonal preconditioner. (a): single-precision performance, (b): double-precision performance.

For run-time performance comparison between GPU and CPU implementations under a variety of conditions, we evaluated the frequency-domain FEM forward model using different mesh complexities. The forward solutions were computed for both a complex-valued problem using a modulation frequency of *ω* = 2*π* · 100 MHz and a real-valued steady-state problem of *ω* = 0. For the complex-valued problem, the BiCGSTAB linear solver was used to compute the linear system in (7) while, for the real-valued problem a CG solver was used. We tested the performance of the GPU solution as a function of the CG and BiCGSTAB convergence tolerances, either without preconditioner or with a diagonal preconditioner. The results are shown in terms of the GPU performance factor, given by the ratio of the CPU and GPU run times.  Figure 6 shows the performance factors for single precision (Figure 6(a)) and double precision (Figure 6(b)) calculations. It can be seen that the GPU achieves a performance factor between 8 and 19 for single precision calculations, depending on the problem type, where the real-valued BiCGSTAB solution without preconditioner shows the highest improvement at 14–19, while the complex BiCGSTAB solution without preconditioner exhibits the smallest improvement at 8–11.5. Generally, the performance factor drops for lower tolerance limits. The performance factors for double-precision solutions are significantly lower, in a range between 3.7 and 4.7. This is due to the fact that while GPU performance drops significantly for double-precision calculations, the CPU solver performance is generally not affected, and indeed the CPU performance is slightly higher at double precision because it avoids casting floating point buffers between single and double precision. The drop in performance factor for lower tolerance limits is not present in the double-precision results.

**Figure 7:**
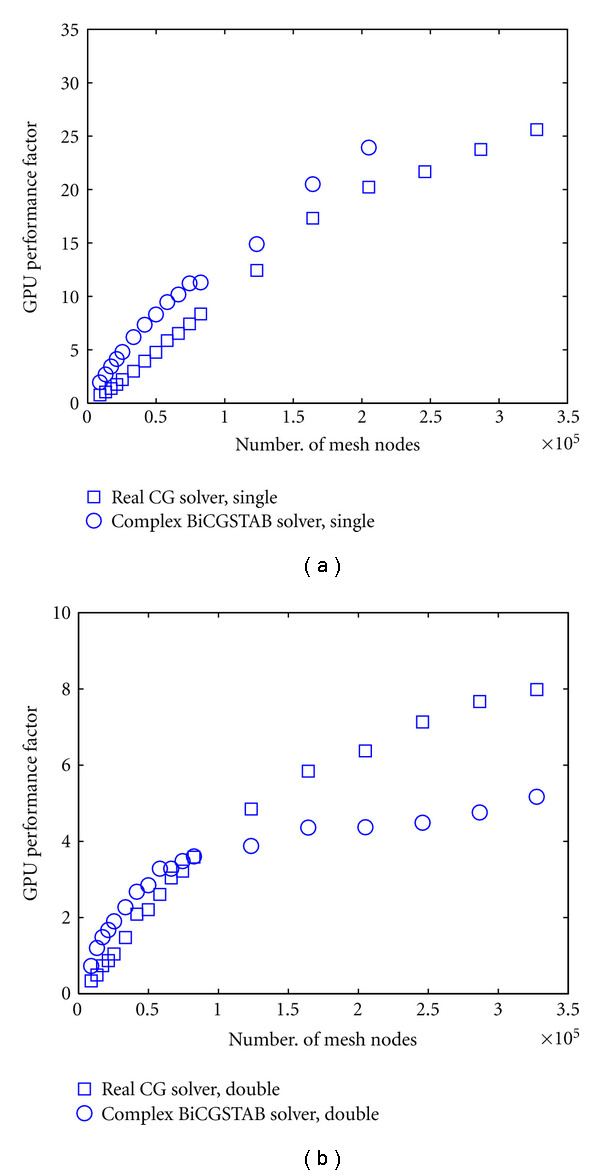
GPU performance factor as a function of mesh node count for a real-valued problem solved with preconditioned CG solver, and a complex-valued problem solved with preconditioned BiCGSTAB solver. (a): single-precision performance, (b): double-precision performance.

The next test compares the CPU and GPU performance as a function of the mesh node density and the resulting size of the linear system. The performance factors for the forward solvers applied to cylindrical meshes of different mesh resolutions as a function of node count are shown in Figure 7. At each mesh resolution, we solved both a real-valued steady-state problem with the preconditioned CG solver, and a complex-valued frequency-domain problem with the preconditioned BiCGSTAB solver, at single-precision (Figure 7(a)) and double-precision (Figure 7(b)) resolution. All solver results are for calculating the real or complex photon density fields for 80 sources, for a solver tolerance fixed at 10^−10^. It can be seen that in all cases GPU performance improves with increasing size of the linear system. For the single-precision solver, the performance factors range between 1 and 26 for mesh node counts between 9000 and 3.3 · 10^5^, respectively, for the steady-state problem, and between 2 and 30 for mesh node counts between 9000 and 2.5 · 10^5^, respectively, for the frequency domain problem. Note that for the frequency domain problem, the performance factors could not be computed for the two largest meshes due to excessive computation time of the CPU solution. The absolute linear solver times for selected cases are shown in Table 2. It can be seen that for the largest mesh resolutions, forward solver times on the CPU can take in excess of an hour. This can be prohibitive for clinical applications in iterative reconstruction, where each step of the reconstruction may require multiple evaluations of the forward problem to calculate the objective function and its gradient at the current estimate or perform a line search along the current search direction. By comparison, the GPU times for these problems typically require 2 to 10 minutes, which is feasible for reconstruction problems.

**Figure 8:**
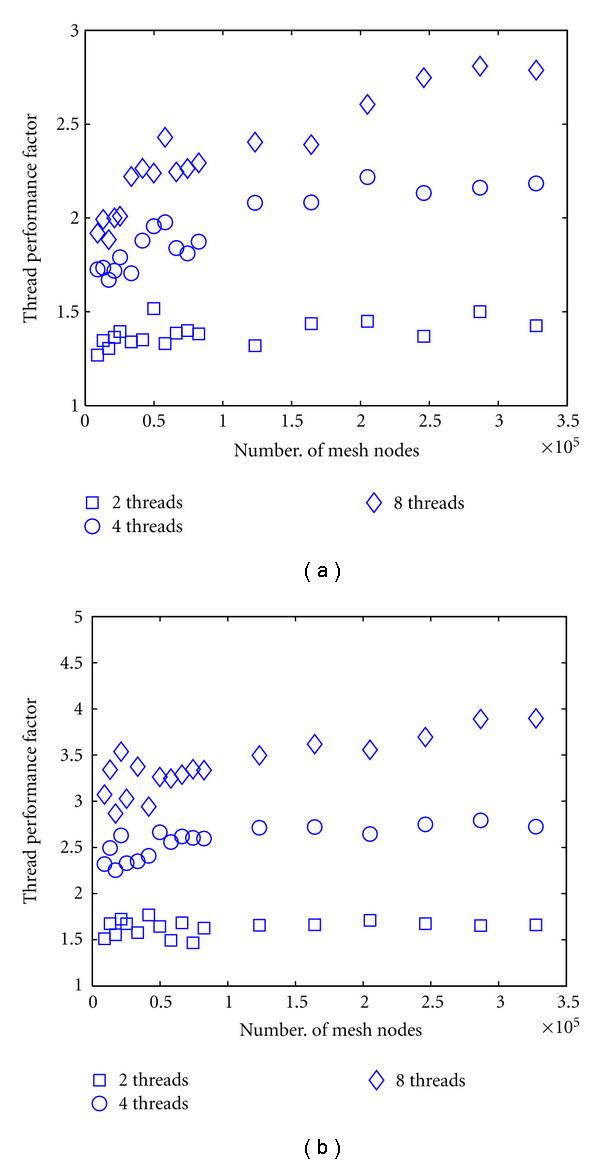
Performance factors for CPU-threaded versus CPU-serial forward solver computations as a function of node densities. (a): CG solver for real-valued problem, (b): BiCGSTAB solver for complex-valued problem.

To provide a comparison with a CPU-based parallel solver, we also show the performance factors of a shared-memory thread-based version of the FEM forward solver using up to 8 threads, compared to the single-thread serial implementation. The thread implementation uses a coarse-grain parallelisation strategy, dividing the solution of the linear problems for different right-hand sides over the available worker threads. This method provided better processor utilisation and less communication overhead for the problem considered here than a fine-grain strategy of parallelising the iterative solver itself. Because the CPU implementation showed no significant performance difference between the single and double precision solution, we present here only the double-precision results.  Figure 8 shows the performance factors for 2, 4, and 8 threads for the real-valued problem using a CG solver, and for the complex-valued problem using a BiCGSTAB solver. The CG solver reaches factors between 1.5 (2 threads) and 2.8 (8 threads) while the BiCGSTAB solver reaches factors between 1.7 (2 threads) and 4 (8 threads). The dependency on mesh complexity is not as marked as for the GPU solver.

### 3.2. Time-Domain Solver

**Figure 9:**
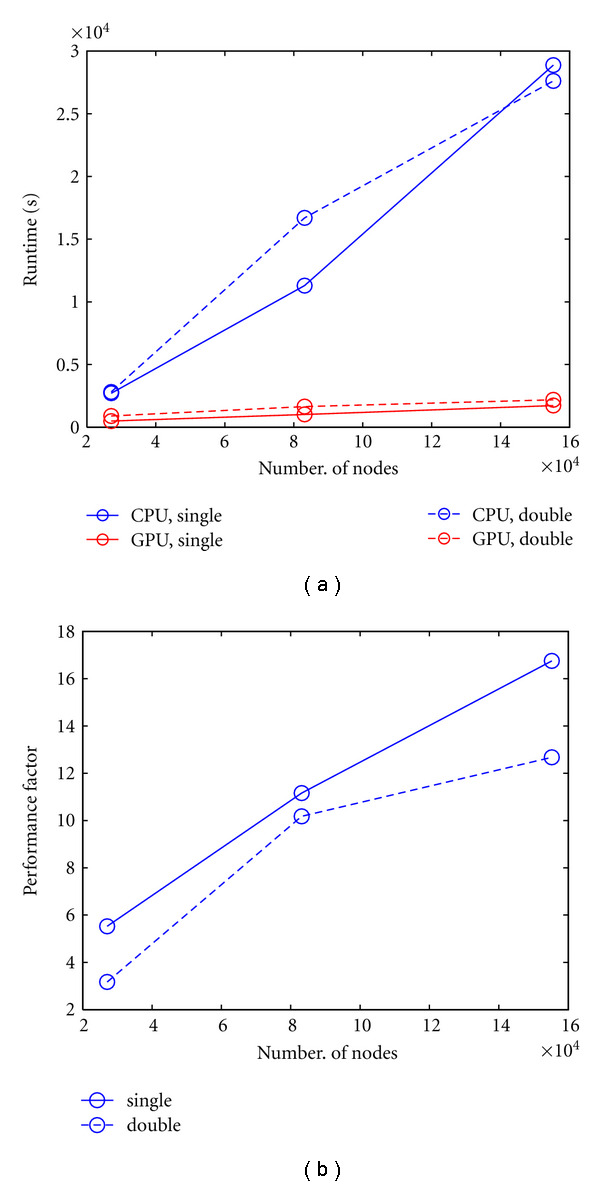
Run-time comparison for CPU and GPU forward solution of time-dependent FEM problem over 100 time steps. (a): Run-times for single and double precision arithmetic as a function of mesh complexity; (b): performance factors.

We computed the finite difference implementation of the time-domain problem (12) over 100 time steps of 50 picoseconds for cylinder meshes of different complexity. For these simulations, a Crank-Nicholson scheme (*θ* = 0.5) was used. Signal intensity time profiles were calculated at 80 detector position for each of 80 source locations. The performance results are shown in Figure 9. It can be seen that the performance improvements of the GPU implementation is again strongly dependent on mesh resolution, ranging from a factor of 3 to 13 for the double-precision arithmetic calculation, and from 6 to 17 for the single-precision calculation. At the highest mesh resolution, the total forward solver run time is approximately 8 hours for the CPU implementation for both single and double precision while the GPU run time is approximately 29 and 36 minutes for the single and double precision solutions, respectively. 

## 4. Conclusions

We have developed a GPU implementation of a finite element forward model for diffuse light transport that can be used as a component in an iterative nonlinear reconstruction method in diffuse optical tomography. The efficiency of the forward solver has a significant impact on reconstruction performance, and the reduction of reconstruction times is essential in making optical tomography a viable imaging modality in clinical diagnosis.

The model presented here supports real and complex-valued problems and can be applied to steady-state, time, or frequency-domain imaging systems. The linear system arising from the FEM discretisation is solved either with a conjugate gradient or biconjugate gradient stabilised iterative solver on the GPU device. We have shown that the GPU solver can achieve significant performance improvements over a serial CPU implementation in the range of factors between 5 and 30, depending on mesh complexity, tolerance limit, and solver type. The GPU-based forward solver provides higher performance gains than a thread-based parallel CPU implementation that was used for comparison. Future developments in GPU hardware are expected to increase the performance gain even further.

We have shown that for the forward problem a single precision linear solver can be applied for typical ranges of optical parameters in clinical applications of optical parameters. Single-precision arithmetic yields higher performance in particular for GPU-computing platforms. However, at very high absorption and scattering parameter values, the linear system may become increasingly ill-conditioned and no longer converge with single-precision arithmetic. In these cases, double-precision computation is required.

## Figures and Tables

**Table 1:** Computational capabilities of GPU platform.

Platform	GeForce GTX 285
Global device memory	1 GB
Processor core clock	1.476 GHz
Memory clock	1.242 GHz
CUDA cores	240
Multiprocessors	30

**Table 2:** GPU run-time comparisons for FEM forward solver computations of 80 source distributions in cylindrical meshes of different node densities. Real-valued problems were solved with a conjugate gradient solver, complex problems with a biconjugate gradient stabilised solver. Values in parentheses are CPU solution times.

Node number	Runtime [s]			
	Real single	Complex single	Real double	Complex double
8987	11.07	13.43	11.1	14.4
	(8.49)	(26.15)	(3.74)	(10.46)
82517	23.24	60.03	26.85	75.42
	(193.94)	(678.65)	(96.21)	(271.9)
245917	82.56	433.89	117.41	523.76
	(1789.7)	(12996.8)	(837.39)	(2351.25)
327617	127.19	1060.42	189.06	909.45
	(3258.46)	(−)	(1509.22)	(4699.71)
